# Supplementary material for: Impact of COVID-19 on Lung Disease in People with Cystic Fibrosis: A 6-Month Follow-Up Study on Respiratory Outcomes
Source: Biomedicines. 2022 Nov 1;10(11):2771. doi: 10.3390/biomedicines10112771 (PMC9687883; doi:10.3390/biomedicines10112771)
Supplement: Supplementary file 1 [file biomedicines-10-02771-s001.zip › Table S1.pdf]

## Online supplements

**Table S1.** Results of chest radiography according to SARS-CoV-2 infectious status.

| | RT-PCR (-) | | RT-PCR (+) | | *P* value[a] |
|---|---|---|---|---|---|
| Patients who received chest x-ray[b] | 29 | 100 | 11 | 100 | |
| Pleural effusion | 3 | 10.3 | 0 | 0 | 0.55 |
| Lobar consolidation | 17 | 58.6 | 2 | 18.2 | 0.022 |
| Interstitial pneumonia | 12 | 41.4 | 9 | 81.8 | 0.022 |
| Pneumothorax | 0 | 0 | 0 | 0 | - |

RT-PCR: Reverse transcriptase-polymerase chain reaction.

[a] Chi-square test or Fisher exact test (when 50% of the cells have expected counts less than 5) for comparison between patients with RT-PCR positive vs negative result

[b] 13 RT-PCR (-) and 15 RT-PCR (+) patients did not receive chest x-ray .
