# Supplementary material for: Impact of COVID-19 on Lung Disease in People with Cystic Fibrosis: A 6-Month Follow-Up Study on Respiratory Outcomes
Source: Biomedicines. 2022 Nov 1;10(11):2771. doi: 10.3390/biomedicines10112771 (PMC9687883; doi:10.3390/biomedicines10112771)
Supplement: Supplementary file 1 [file biomedicines-10-02771-s001.zip › Table S2.pdf]

## Online Supplement

**Table S2.** Sputum microbiology at the time of RT-PCR (baseline) and at 6-month follow-up according to SARS-CoV-2 infectious status.

| | RT-PCR (-) (N= 31)[a] | | RT-PCR (+) (N= 23)[a] | | *p*-value[b] |
|---|---|---|---|---|---|
| | Baseline | 6 months | Baseline | 6 months | |
| P. *aeruginosa* | 21 (67.7) | 18 (58.1) | 13 (56.5) | 12 (52.2) | 0.82 |
| A. *xylosoxidans* | 5 (16.1) | 4 (12.9) | 4 (17.4) | 1 (4.3) | - |
| S. *maltophilia* | 4 (12.9) | 1 (3.2) | 2 (8.7) | 0 | - |
| MRSA | 4 (12.9) | 4 (12.9) | 5 (21.7) | 3 (13.0) | - |
| B. *cepacia* complex | 1 (3.2) | 1 (3.2) | 0 | 0 | - |
| NTM | 2 (6.5) | 3 (9.7) | 2 (8.7) | 1 (4.3) | - |
| *Aspergiullus* spp | 12 (38.7) | 4 (12.9) | 4 (17.4) | 2 (8.7) | - |

MRSA: Methicillin-resistant *Staphylococcus aureus*. NTM: Nontuberculous mycobacteria.

[a] The table gives the number of positive sputum cultures among patients with available follow-up data (31 out of 42 RT-PCR negative and 23 out of 24 RT-PCR positive patients).

[b] Differences in prevalence of positive sputum culture for P. *aeruginosa* at 6 months were tested in a logistic regression model including terms for baseline infectious status and group (RT-PCR positive vs RT-PCR-negative). Differences in other respiratory infections were not tested due to the limited numbers in each group.
